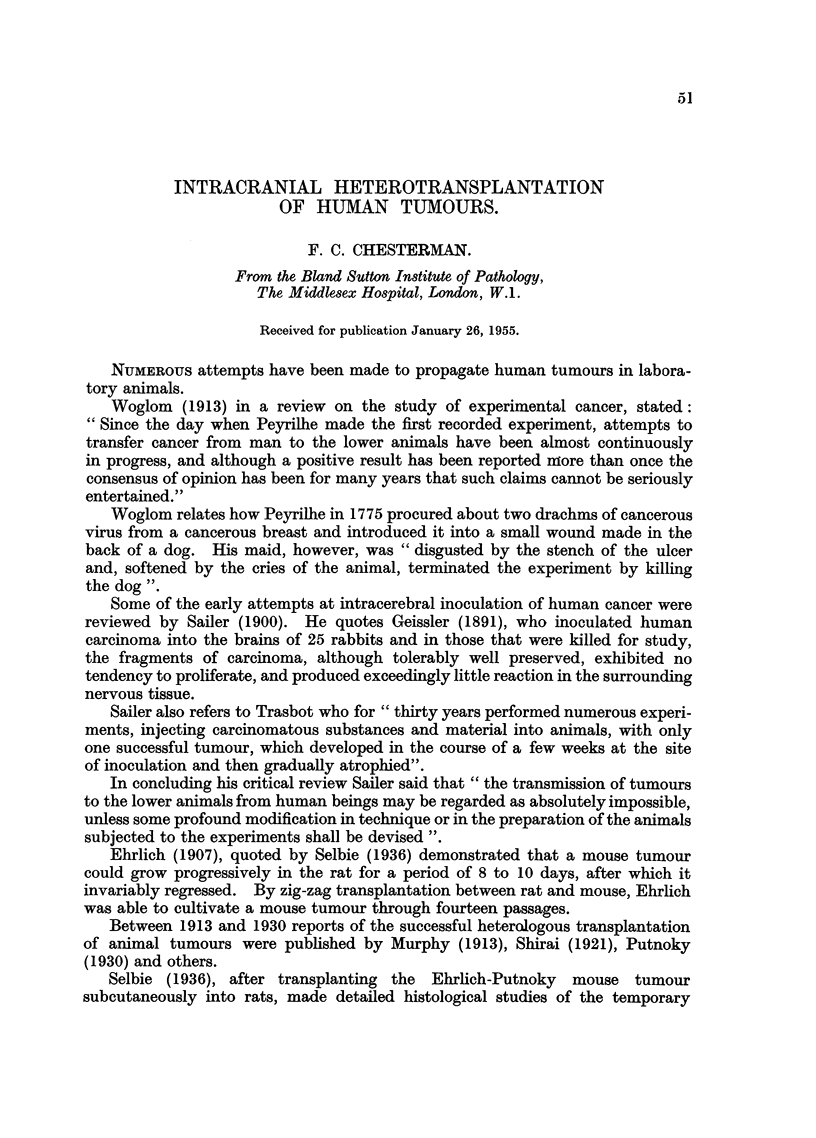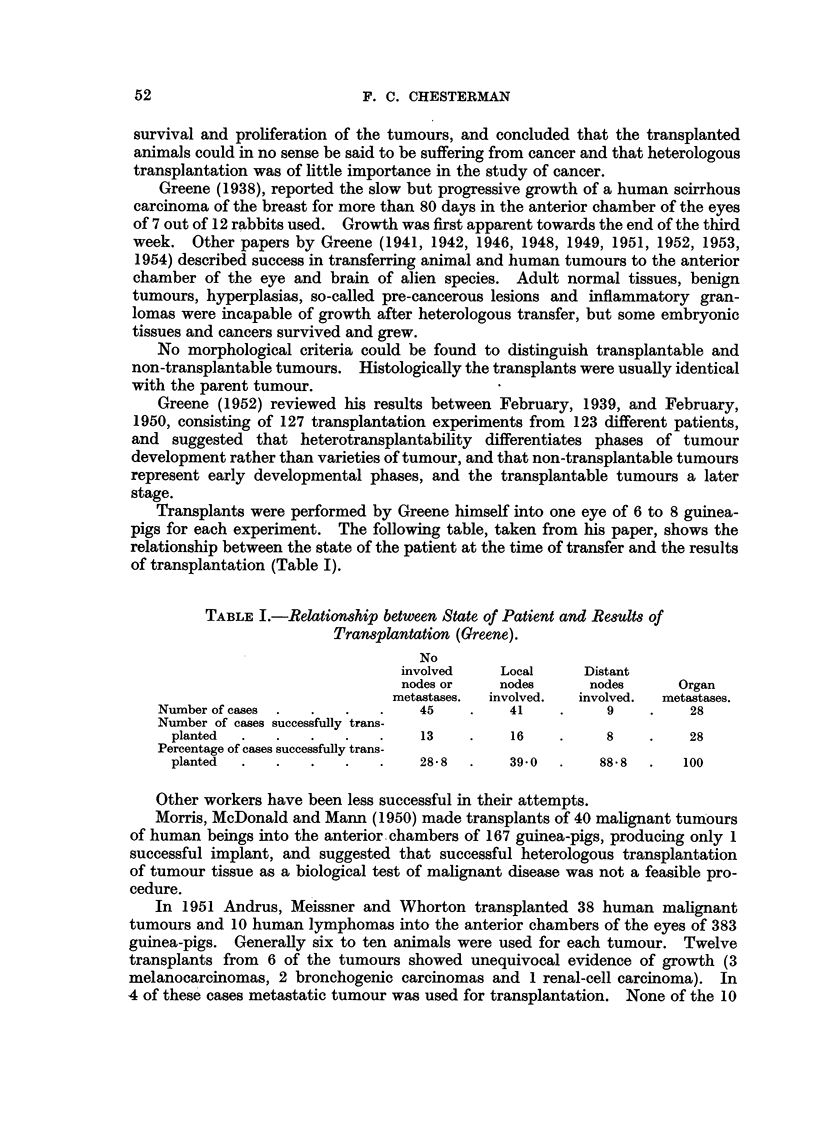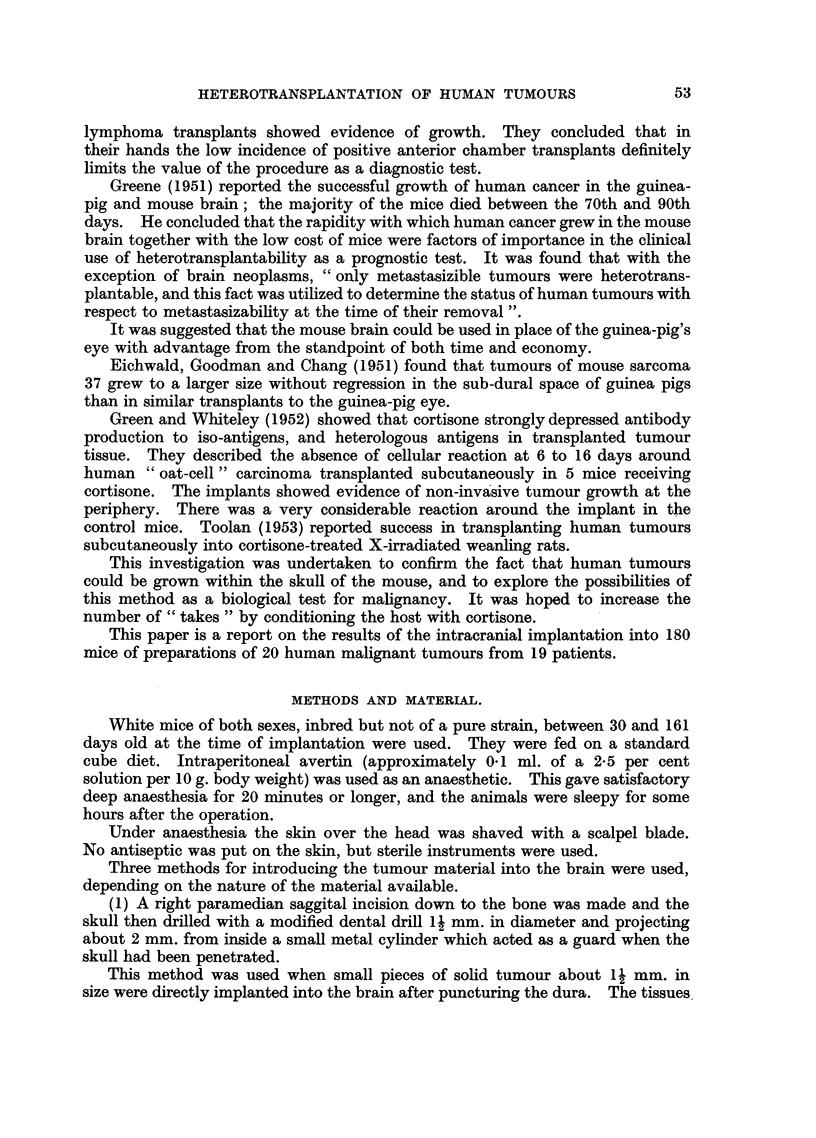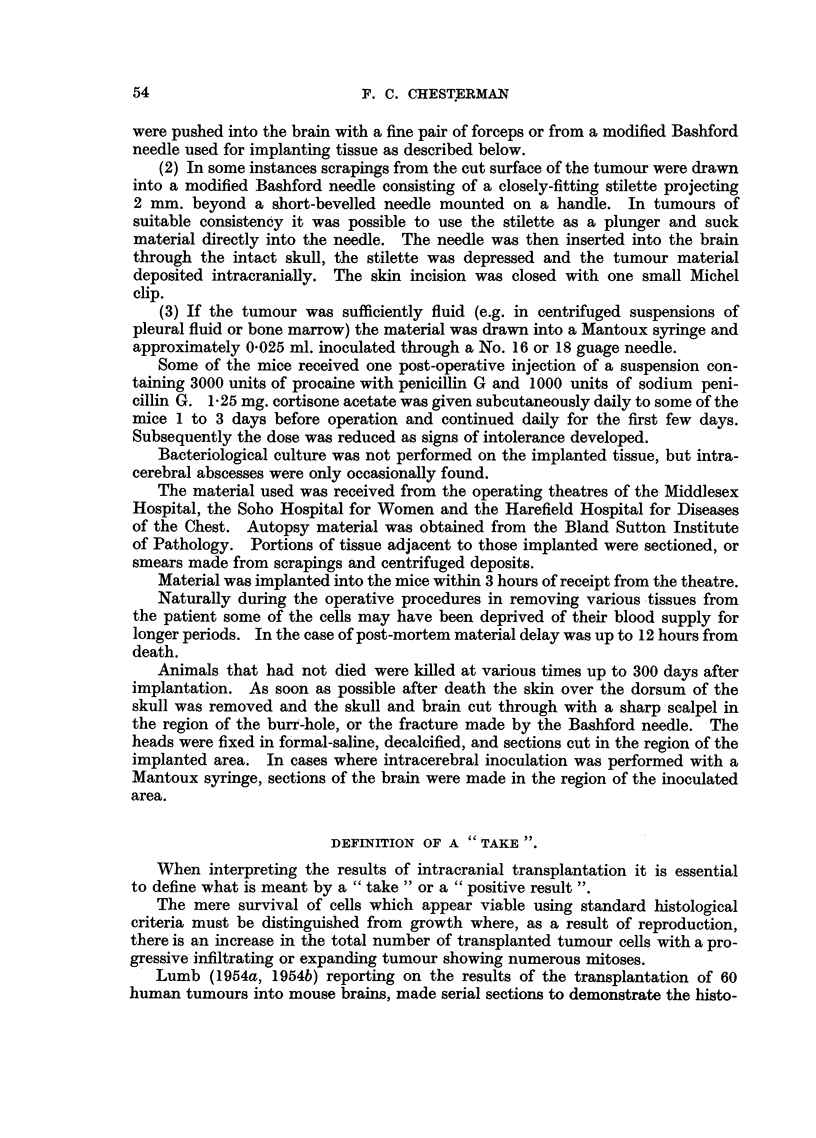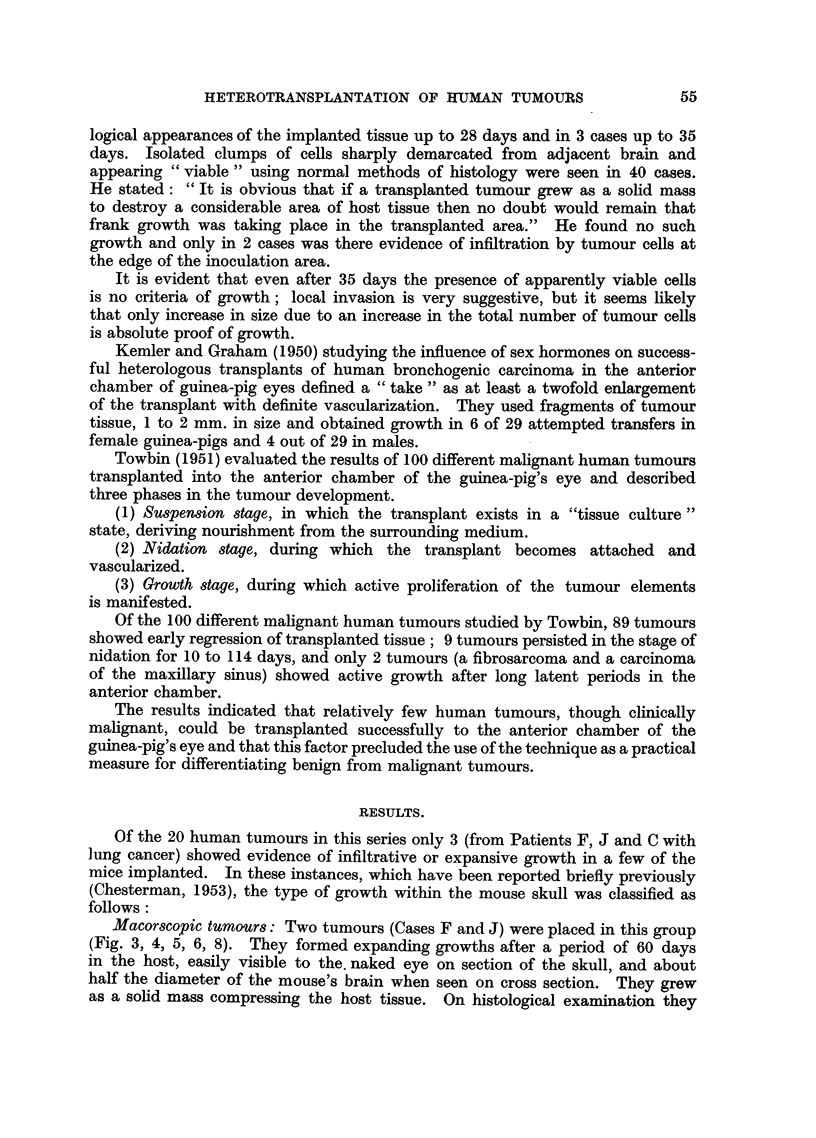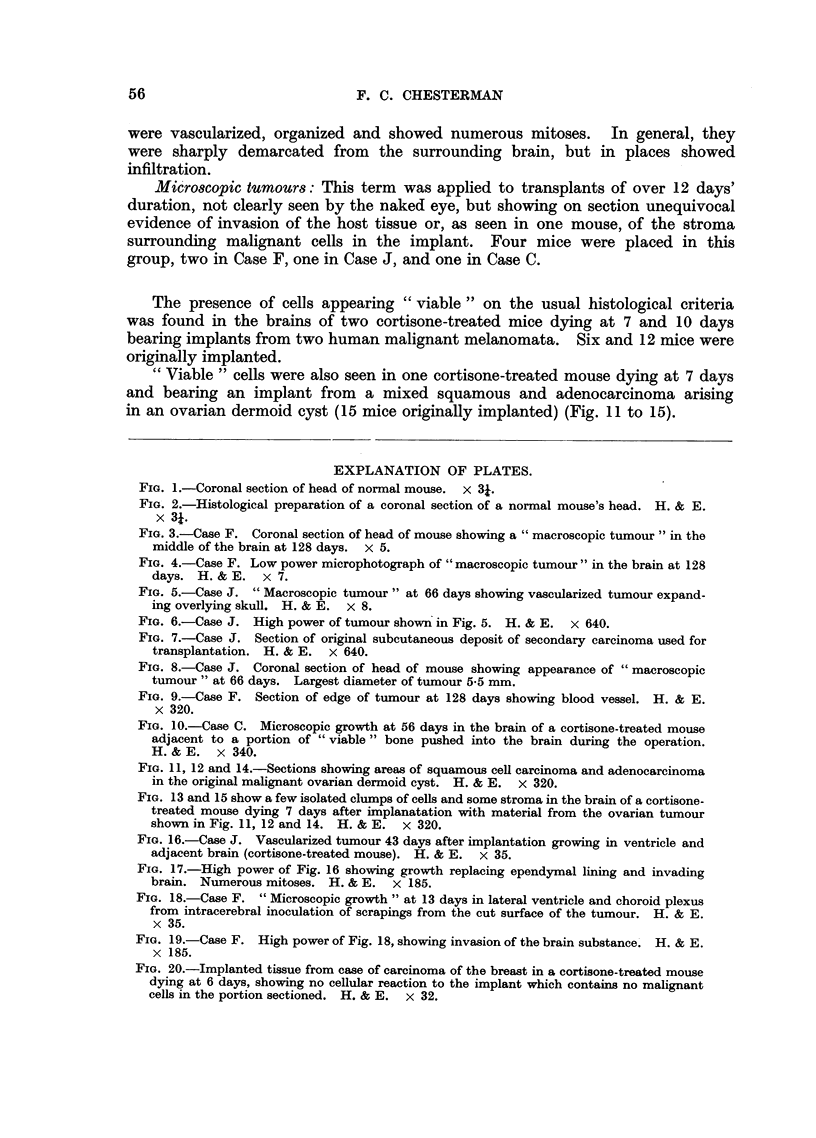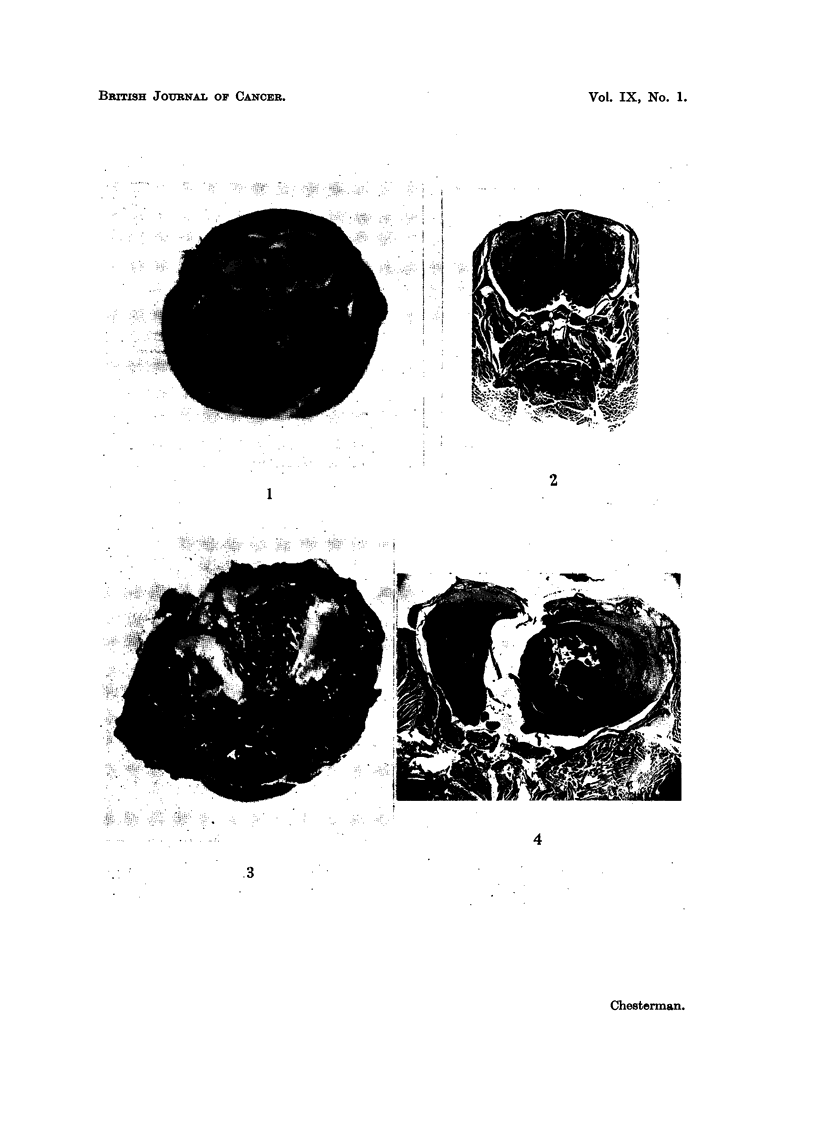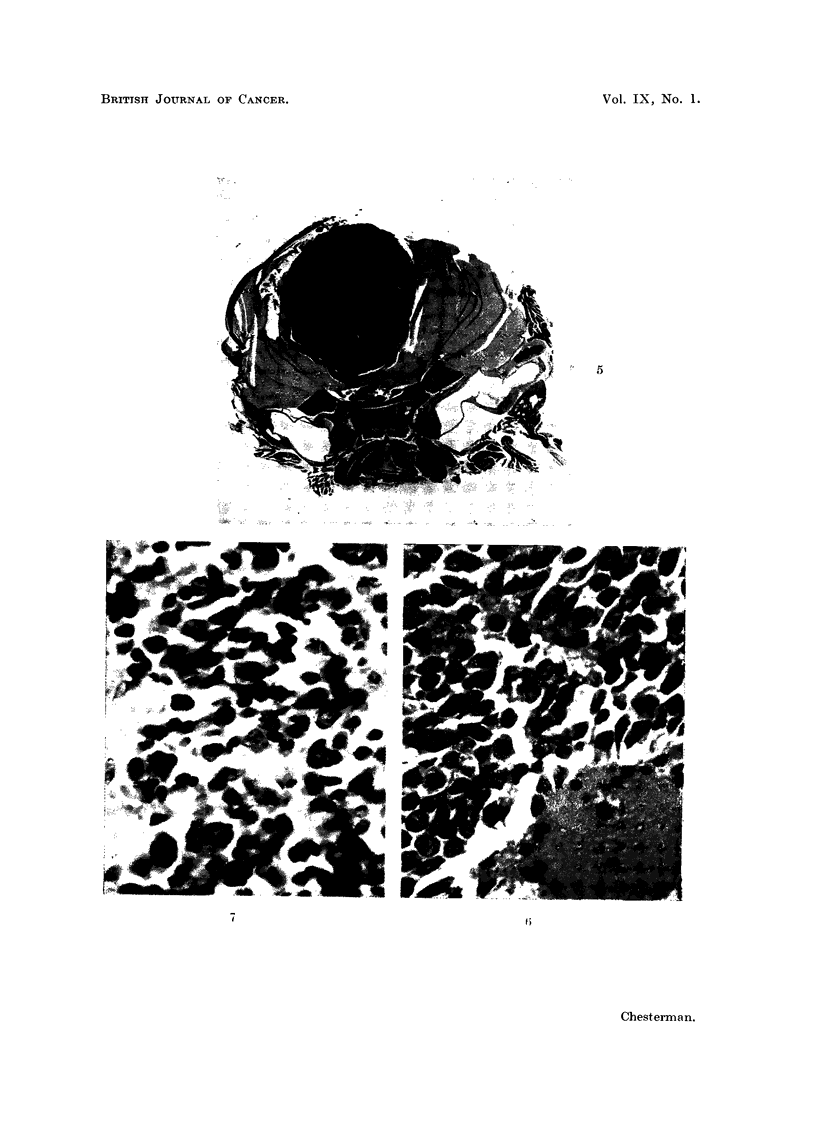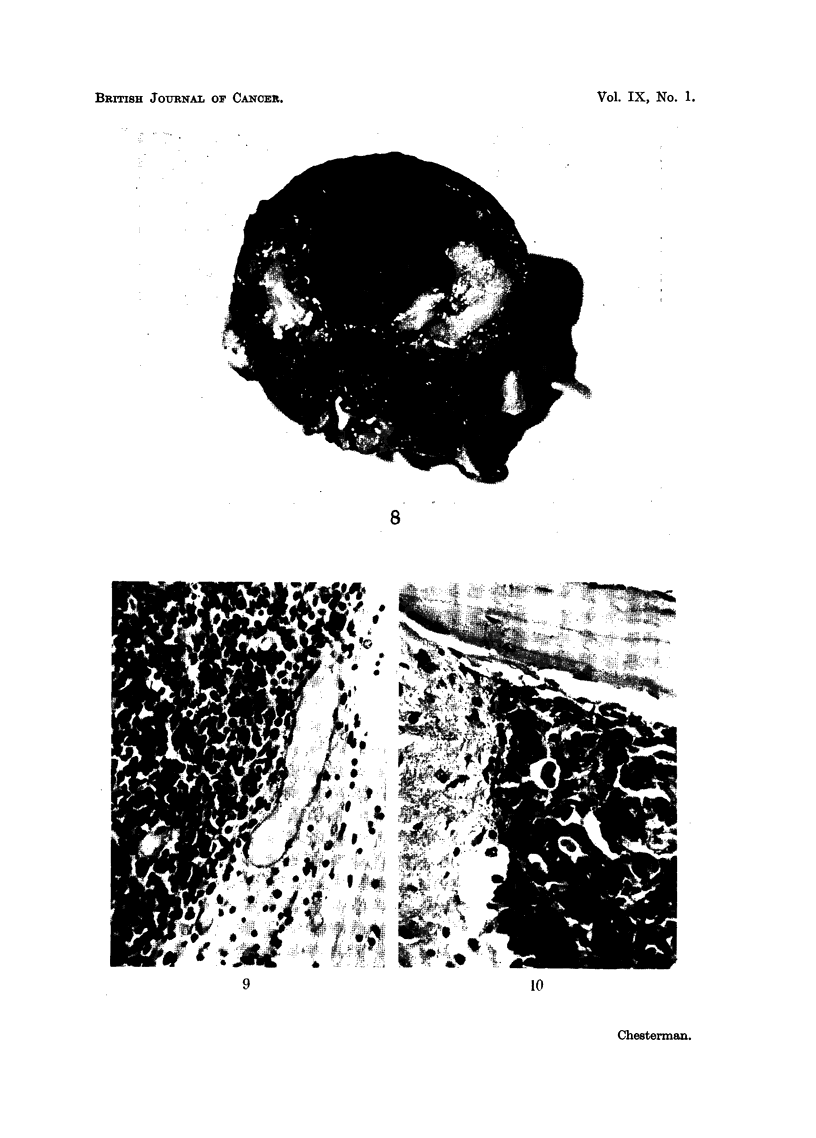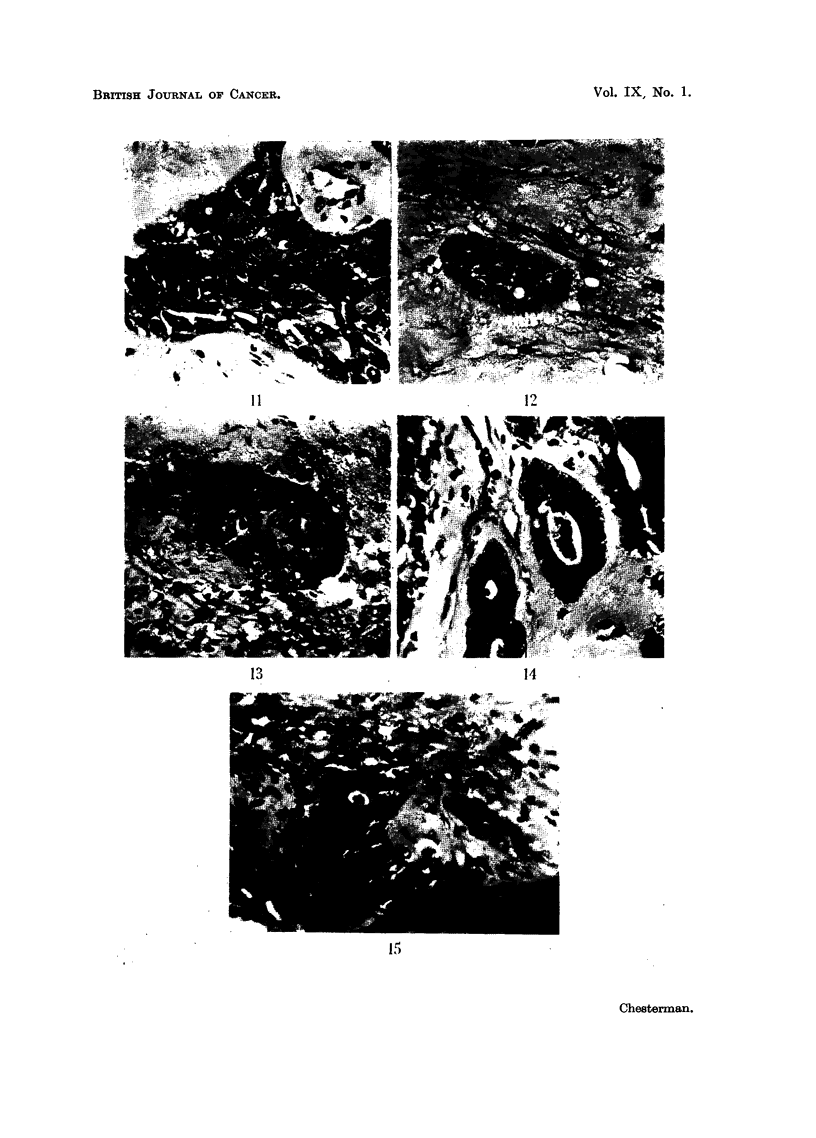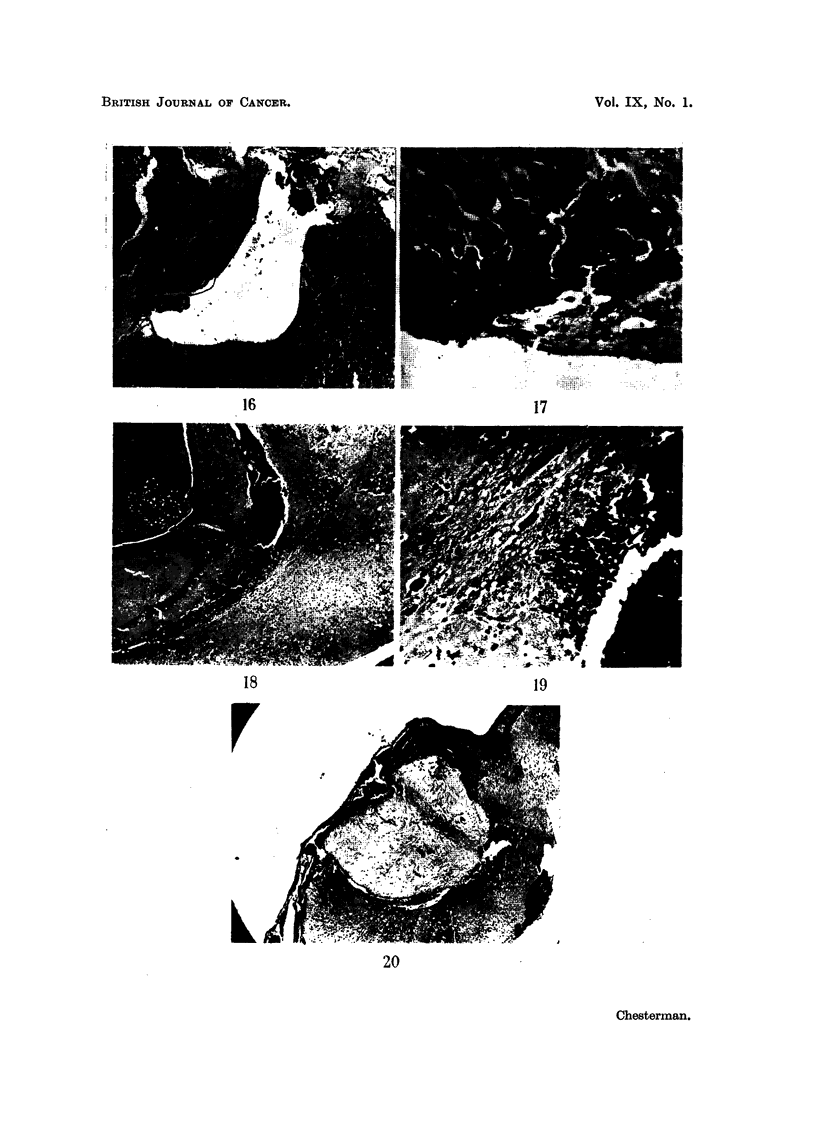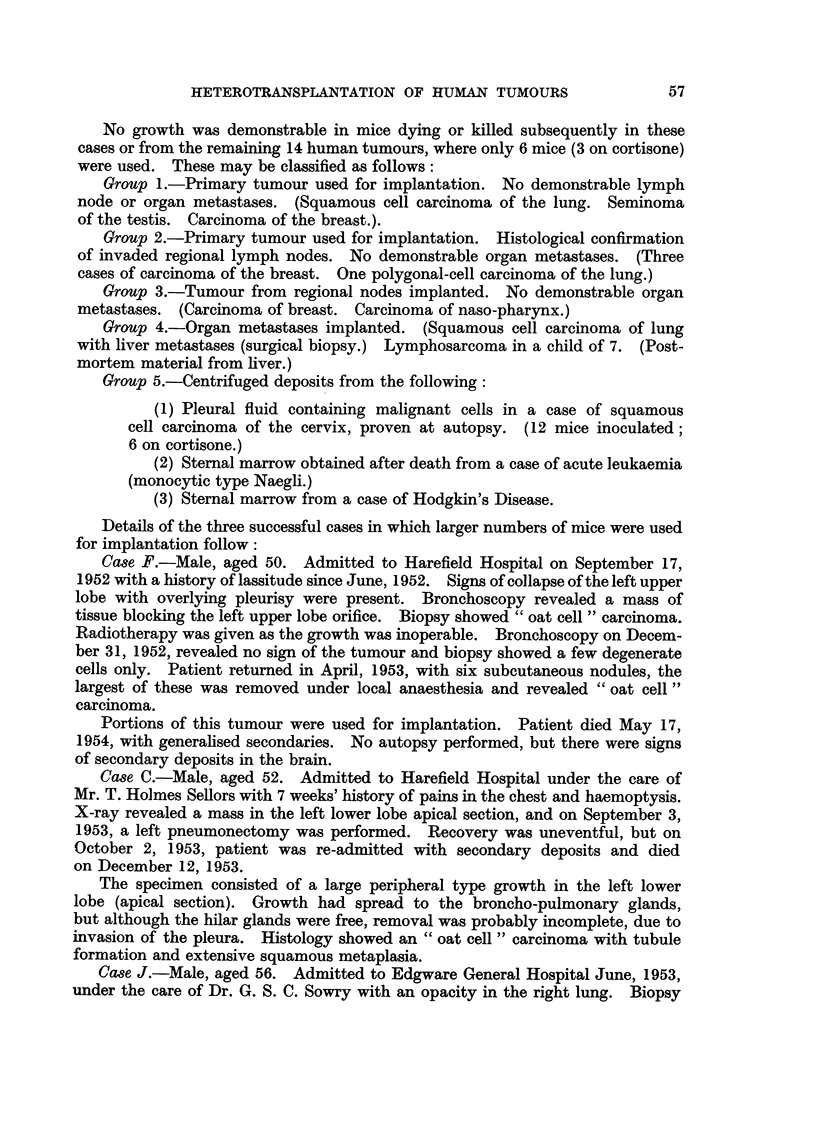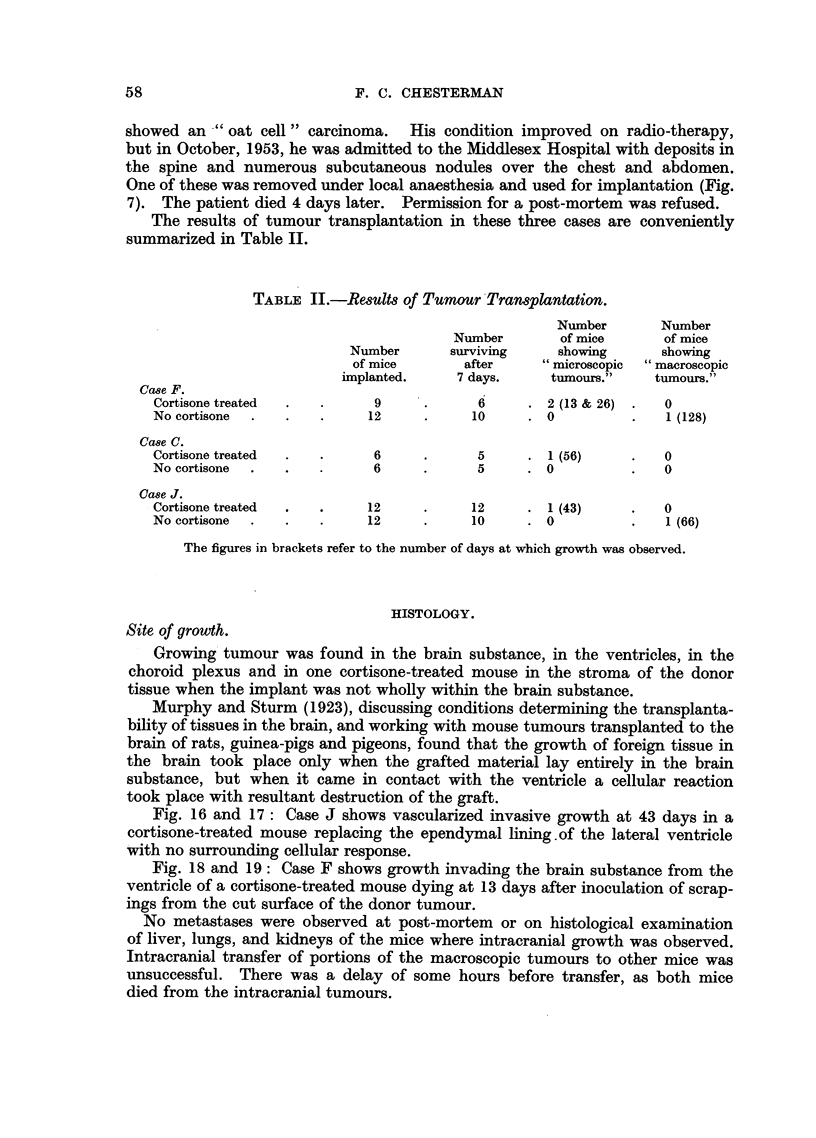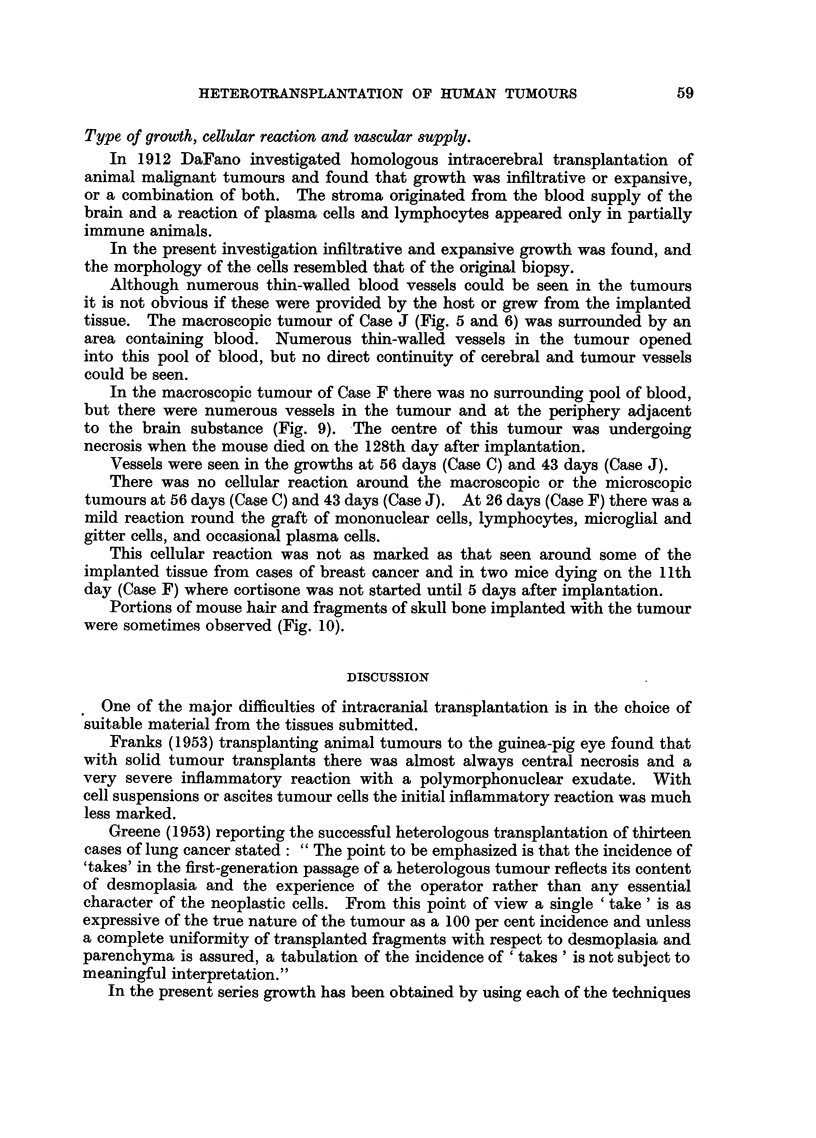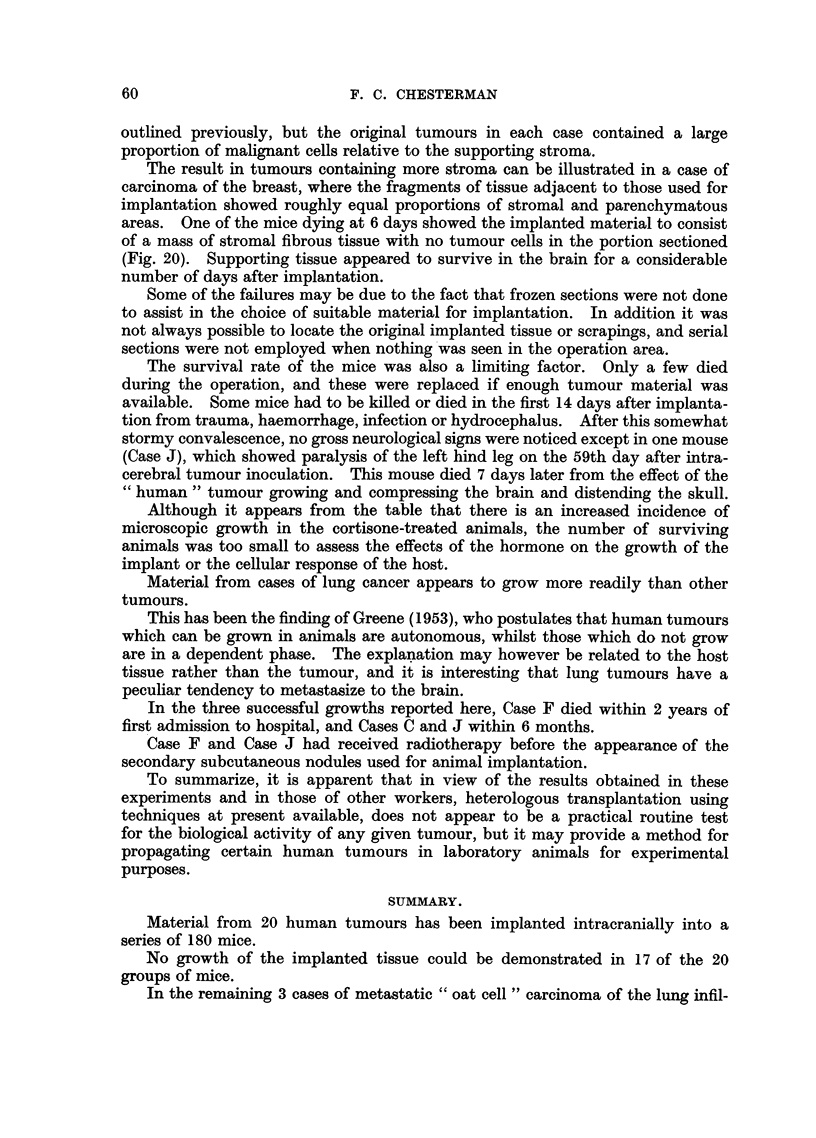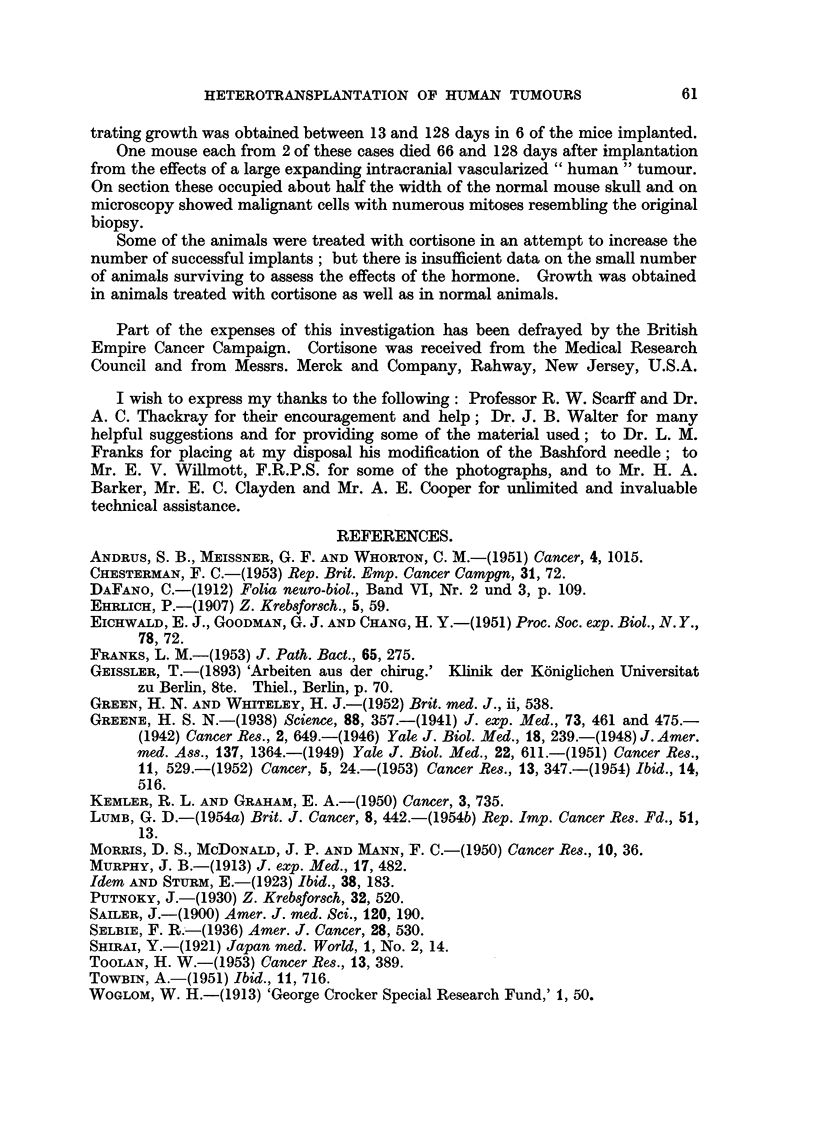# Intracranial Heterotransplantation of Human Tumours

**DOI:** 10.1038/bjc.1955.5

**Published:** 1955-03

**Authors:** F. C. Chesterman

## Abstract

**Images:**


### INTRACRANIAL HETEROTRANSPLANTATION OF HUMAN TUMOURS.

F. C. CHESTERMAN.

*From the Bland Sutton Institute of Pathology, The Middlesex Hospital, London, W.*1.

Received for publication January 26, 1955.

NUMEROUS attempts have been made to propagate human tumours in laboratory animals.

Woglom (1913) in a review on the study of experimental cancer, stated: " Since the day when Peyrilhe made the first recorded experiment, attempts to transfer cancer from man to the lower animals have been almost continuously in progress, and although a positive result has been reported more than once the consensus of opinion has been for many years that such claims cannot be seriously entertained."

Woglom relates how Peyrilhe in 1775 procured about two drachms of cancerous virus from a cancerous breast and introduced it into a small wound made in the back of a dog. His maid, however, was " disgusted by the stench of the ulcer and, softened by the cries of the animal, terminated the experiment by killing the dog ".

Some of the early attempts at intracerebral inoculation of human cancer were reviewed by Sailer (1900). He quotes Geissler (1891), who inoculated human carcinoma into the brains of 25 rabbits and in those that were killed for study, the fragments of carcinoma, although tolerably well preserved, exhibited no tendency to proliferate, and produced exceedingly little reaction in the surrounding nervous tissue.

Sailer also refers to Trasbot who for " thirty years performed numerous experiments, injecting carcinomatous substances and material into animals, with only one successful tumour, which developed in the course of a few weeks at the site of inoculation and then gradually atrophied".

In concluding his critical review Sailer said that " the transmission of tumours to the lower animals from human beings may be regarded as absolutely impossible, unless some profound modification in technique or in the preparation of the animals subjected to the experiments shall be devised ".

Ehrlich (1907), quoted by Selbie (1936) demonstrated that a mouse tumour could grow progressively in the rat for a period of 8 to 10 days, after which it invariably regressed. By zig-zag transplantation between rat and mouse, Ehrlich was able to cultivate a mouse tumour through fourteen passages.

Between 1913 and 1930 reports of the successful heterologous transplantation of animal tumours were published by Murphy (1913), Shirai (1921), Putnoky (1930) and others.

Selbie (1936), after transplanting the Ehrlich-Putnoky mouse tumour subcutaneously into rats, made detailed histological studies of the temporary survival and proliferation of the tumours, and concluded that the transplanted animals could in no sense be said to be suffering from cancer and that heterologous transplantation was of little importance in the study of cancer.

Greene (1938), reported the slow but progressive growth of a human scirrhous carcinoma of the breast for more than 80 days in the anterior chamber of the eyes of 7 out of 12 rabbits used. Growth was first apparent towards the end of the third week. Other papers by Greene (1941, 1942, 1946, 1948, 1949, 1951, 1952, 1953, 1954) described success in transferring animal and human tumours to the anterior chamber of the eye and brain of alien species. Adult normal tissues, benign tumours, hyperplasias, so-called pre-cancerous lesions and inflammatory granlomas were incapable of growth after heterologous transfer, but some embryonic tissues and cancers survived and grew.

No morphological criteria could be found to distinguish transplantable and non-transplantable tumours. Histologically the transplants were usually identical with the parent tumour.

Greene (1952) reviewed his results between February, 1939, and February, 1950, consisting of 127 transplantation experiments from 123 different patients, and suggested that heterotransplantability differentiates phases of tumour development rather than varieties of tumour, and that non-transplantable tumours represent early developmental phases, and the transplantable tumours a later stage.

Transplants were performed by Greene himself into one eye of 6 to 8 guinea-pigs for each experiment. The following table, taken from his paper, shows the relationship between the state of the patient at the time of transfer and the results of transplantation (Table I).

TABLE I.—*Relationship between State of Patient and Results of Transplantation (Greene).*

| | No involved nodes or metastases. | Local nodes involved. | Distant nodes involved. | Organ metastases. |
|---|---|---|---|---|
| Number of cases | 45 | 41 | 9 | 28 |
| Number of cases successfully transplanted | 13 | 16 | 8 | 28 |
| Percentage of cases successfully transplanted | 28·8 | 39·0 | 88·8 | 100 |

Other workers have been less successful in their attempts.

Morris, McDonald and Mann (1950) made transplants of 40 malignant tumours of human beings into the anterior chambers of 167 guinea-pigs, producing only 1 successful implant, and suggested that successful heterologous transplantation of tumour tissue as a biological test of malignant disease was not a feasible procedure.

In 1951 Andrus, Meissner and Whorton transplanted 38 human malignant tumours and 10 human lymphomas into the anterior chambers of the eyes of 383 guinea-pigs. Generally six to ten animals were used for each tumour. Twelve transplants from 6 of the tumours showed unequivocal evidence of growth (3 melanocarcinomas, 2 bronchogenic carcinomas and 1 renal-cell carcinoma). In 4 of these cases metastatic tumour was used for transplantation. None of the 10

lymphoma transplants showed evidence of growth. They concluded that in their hands the low incidence of positive anterior chamber transplants definitely limits the value of the procedure as a diagnostic test.

Greene (1951) reported the successful growth of human cancer in the guinea-pig and mouse brain ; the majority of the mice died between the 70th and 90th days. He concluded that the rapidity with which human cancer grew in the mouse brain together with the low cost of mice were factors of importance in the clinical use of heterotransplantability as a prognostic test. It was found that with the exception of brain neoplasms, " only metastasizible tumours were heterotransplantable, and this fact was utilized to determine the status of human tumours with respect to metastasizability at the time of their removal ".

It was suggested that the mouse brain could be used in place of the guinea-pig's eye with advantage from the standpoint of both time and economy.

Eichwald, Goodman and Chang (1951) found that tumours of mouse sarcoma 37 grew to a larger size without regression in the sub-dural space of guinea pigs than in similar transplants to the guinea-pig eye.

Green and Whiteley (1952) showed that cortisone strongly depressed antibody production to iso-antigens, and heterologous antigens in transplanted tumour tissue. They described the absence of cellular reaction at 6 to 16 days around human " oat-cell " carcinoma transplanted subcutaneously in 5 mice receiving cortisone. The implants showed evidence of non-invasive tumour growth at the periphery. There was a very considerable reaction around the implant in the control mice. Toolan (1953) reported success in transplanting human tumours subcutaneously into cortisone-treated X-irradiated weanling rats.

This investigation was undertaken to confirm the fact that human tumours could be grown within the skull of the mouse, and to explore the possibilities of this method as a biological test for malignancy. It was hoped to increase the number of " takes " by conditioning the host with cortisone.

This paper is a report on the results of the intracranial implantation into 180 mice of preparations of 20 human malignant tumours from 19 patients.

### METHODS AND MATERIAL.

White mice of both sexes, inbred but not of a pure strain, between 30 and 161 days old at the time of implantation were used. They were fed on a standard cube diet. Intraperitoneal avertin (approximately 0·1 ml. of a 2·5 per cent solution per 10 g. body weight) was used as an anaesthetic. This gave satisfactory deep anaesthesia for 20 minutes or longer, and the animals were sleepy for some hours after the operation.

Under anaesthesia the skin over the head was shaved with a scalpel blade. No antiseptic was put on the skin, but sterile instruments were used.

Three methods for introducing the tumour material into the brain were used, depending on the nature of the material available.

(1) A right paramedian saggital incision down to the bone was made and the skull then drilled with a modified dental drill 1½ mm. in diameter and projecting about 2 mm. from inside a small metal cylinder which acted as a guard when the skull had been penetrated.

This method was used when small pieces of solid tumour about 1½ mm. in size were directly implanted into the brain after puncturing the dura. The tissues were pushed into the brain with a fine pair of forceps or from a modified Bashford needle used for implanting tissue as described below.

(2) In some instances scrapings from the cut surface of the tumour were drawn into a modified Bashford needle consisting of a closely-fitting stilette projecting 2 mm. beyond a short-bevelled needle mounted on a handle. In tumours of suitable consistency it was possible to use the stilette as a plunger and suck material directly into the needle. The needle was then inserted into the brain through the intact skull, the stilette was depressed and the tumour material deposited intracranially. The skin incision was closed with one small Michel clip.

(3) If the tumour was sufficiently fluid (e.g. in centrifuged suspensions of pleural fluid or bone marrow) the material was drawn into a Mantoux syringe and approximately 0·025 ml. inoculated through a No. 16 or 18 guage needle.

Some of the mice received one post-operative injection of a suspension containing 3000 units of procaine with penicillin G and 1000 units of sodium penicillin G. 1·25 mg. cortisone acetate was given subcutaneously daily to some of the mice 1 to 3 days before operation and continued daily for the first few days. Subsequently the dose was reduced as signs of intolerance developed.

Bacteriological culture was not performed on the implanted tissue, but intracerebral abscesses were only occasionally found.

The material used was received from the operating theatres of the Middlesex Hospital, the Soho Hospital for Women and the Harefield Hospital for Diseases of the Chest. Autopsy material was obtained from the Bland Sutton Institute of Pathology. Portions of tissue adjacent to those implanted were sectioned, or smears made from scrapings and centrifuged deposits.

Material was implanted into the mice within 3 hours of receipt from the theatre.

Naturally during the operative procedures in removing various tissues from the patient some of the cells may have been deprived of their blood supply for longer periods. In the case of post-mortem material delay was up to 12 hours from death.

Animals that had not died were killed at various times up to 300 days after implantation. As soon as possible after death the skin over the dorsum of the skull was removed and the skull and brain cut through with a sharp scalpel in the region of the burr-hole, or the fracture made by the Bashford needle. The heads were fixed in formal-saline, decalcified, and sections cut in the region of the implanted area. In cases where intracerebral inoculation was performed with a Mantoux syringe, sections of the brain were made in the region of the inoculated area.

### DEFINITION OF A " TAKE ".

When interpreting the results of intracranial transplantation it is essential to define what is meant by a " take " or a " positive result ".

The mere survival of cells which appear viable using standard histological criteria must be distinguished from growth where, as a result of reproduction, there is an increase in the total number of transplanted tumour cells with a progressive infiltrating or expanding tumour showing numerous mitoses.

Lumb (1954*a*, 1954*b*) reporting on the results of the transplantation of 60 human tumours into mouse brains, made serial sections to demonstrate the histological appearances of the implanted tissue up to 28 days and in 3 cases up to 35 days. Isolated clumps of cells sharply demarcated from adjacent brain and appearing " viable " using normal methods of histology were seen in 40 cases. He stated : " It is obvious that if a transplanted tumour grew as a solid mass to destroy a considerable area of host tissue then no doubt would remain that frank growth was taking place in the transplanted area." He found no such growth and only in 2 cases was there evidence of infiltration by tumour cells at the edge of the inoculation area.

It is evident that even after 35 days the presence of apparently viable cells is no criteria of growth ; local invasion is very suggestive, but it seems likely that only increase in size due to an increase in the total number of tumour cells is absolute proof of growth.

Kemler and Graham (1950) studying the influence of sex hormones on successful heterologous transplants of human bronchogenic carcinoma in the anterior chamber of guinea-pig eyes defined a " take " as at least a twofold enlargement of the transplant with definite vascularization. They used fragments of tumour tissue, 1 to 2 mm. in size and obtained growth in 6 of 29 attempted transfers in female guinea-pigs and 4 out of 29 in males.

Towbin (1951) evaluated the results of 100 different malignant human tumours transplanted into the anterior chamber of the guinea-pig's eye and described three phases in the tumour development.

(1) *Suspension stage*, in which the transplant exists in a "tissue culture " state, deriving nourishment from the surrounding medium.

(2) *Nidation stage*, during which the transplant becomes attached and vascularized.

(3) *Growth stage*, during which active proliferation of the tumour elements is manifested.

Of the 100 different malignant human tumours studied by Towbin, 89 tumours showed early regression of transplanted tissue ; 9 tumours persisted in the stage of nidation for 10 to 114 days, and only 2 tumours (a fibrosarcoma and a carcinoma of the maxillary sinus) showed active growth after long latent periods in the anterior chamber.

The results indicated that relatively few human tumours, though clinically malignant, could be transplanted successfully to the anterior chamber of the guinea-pig's eye and that this factor precluded the use of the technique as a practical measure for differentiating benign from malignant tumours.

### RESULTS.

Of the 20 human tumours in this series only 3 (from Patients F, J and C with lung cancer) showed evidence of infiltrative or expansive growth in a few of the mice implanted. In these instances, which have been reported briefly previously (Chesterman, 1953), the type of growth within the mouse skull was classified as follows :

*Macorscopic tumours :* Two tumours (Cases F and J) were placed in this group (Fig. 3, 4, 5, 6, 8). They formed expanding growths after a period of 60 days in the host, easily visible to the naked eye on section of the skull, and about half the diameter of the mouse's brain when seen on cross section. They grew as a solid mass compressing the host tissue. On histological examination they were vascularized, organized and showed numerous mitoses. In general, they were sharply demarcated from the surrounding brain, but in places showed infiltration.

*Microscopic tumours:* This term was applied to transplants of over 12 days' duration, not clearly seen by the naked eye, but showing on section unequivocal evidence of invasion of the host tissue or, as seen in one mouse, of the stroma surrounding malignant cells in the implant. Four mice were placed in this group, two in Case F, one in Case J, and one in Case C.

The presence of cells appearing "viable" on the usual histological criteria was found in the brains of two cortisone-treated mice dying at 7 and 10 days bearing implants from two human malignant melanomata. Six and 12 mice were originally implanted.

"Viable" cells were also seen in one cortisone-treated mouse dying at 7 days and bearing an implant from a mixed squamous and adenocarcinoma arising in an ovarian dermoid cyst (15 mice originally implanted) (Fig. 11 to 15).

---

### EXPLANATION OF PLATES.

FIG. 1.—Coronal section of head of normal mouse. × 3¼.

FIG. 2.—Histological preparation of a coronal section of a normal mouse's head. H. & E. × 3¼.

FIG. 3.—Case F. Coronal section of head of mouse showing a "macroscopic tumour" in the middle of the brain at 128 days. × 5.

FIG. 4.—Case F. Low power microphotograph of "macroscopic tumour" in the brain at 128 days. H. & E. × 7.

FIG. 5.—Case J. "Macroscopic tumour" at 66 days showing vascularized tumour expanding overlying skull. H. & E. × 8.

FIG. 6.—Case J. High power of tumour shown in Fig. 5. H. & E. × 640.

FIG. 7.—Case J. Section of original subcutaneous deposit of secondary carcinoma used for transplantation. H. & E. × 640.

FIG. 8.—Case J. Coronal section of head of mouse showing appearance of "macroscopic tumour" at 66 days. Largest diameter of tumour 5·5 mm.

FIG. 9.—Case F. Section of edge of tumour at 128 days showing blood vessel. H. & E. × 320.

FIG. 10.—Case C. Microscopic growth at 56 days in the brain of a cortisone-treated mouse adjacent to a portion of "viable" bone pushed into the brain during the operation. H. & E. × 340.

FIG. 11, 12 and 14.—Sections showing areas of squamous cell carcinoma and adenocarcinoma in the original malignant ovarian dermoid cyst. H. & E. × 320.

FIG. 13 and 15 show a few isolated clumps of cells and some stroma in the brain of a cortisone-treated mouse dying 7 days after implanatation with material from the ovarian tumour shown in Fig. 11, 12 and 14. H. & E. × 320.

FIG. 16.—Case J. Vascularized tumour 43 days after implantation growing in ventricle and adjacent brain (cortisone-treated mouse). H. & E. × 35.

FIG. 17.—High power of Fig. 16 showing growth replacing ependymal lining and invading brain. Numerous mitoses. H. & E. × 185.

FIG. 18.—Case F. "Microscopic growth" at 13 days in lateral ventricle and choroid plexus from intracerebral inoculation of scrapings from the cut surface of the tumour. H. & E. × 35.

FIG. 19.—Case F. High power of Fig. 18, showing invasion of the brain substance. H. & E. × 185.

FIG. 20.—Implanted tissue from case of carcinoma of the breast in a cortisone-treated mouse dying at 6 days, showing no cellular reaction to the implant which contains no malignant cells in the portion sectioned. H. & E. × 32.

1

2

3

4

Chesterman.

5

7

6

Chesterman.

8

9

10

Chesterman.

11

12

13

14

15

Chesterman.

16

17

18

19

20

No growth was demonstrable in mice dying or killed subsequently in these cases or from the remaining 14 human tumours, where only 6 mice (3 on cortisone) were used. These may be classified as follows :

*Group* 1.—Primary tumour used for implantation. No demonstrable lymph node or organ metastases. (Squamous cell carcinoma of the lung. Seminoma of the testis. Carcinoma of the breast.).

*Group* 2.—Primary tumour used for implantation. Histological confirmation of invaded regional lymph nodes. No demonstrable organ metastases. (Three cases of carcinoma of the breast. One polygonal-cell carcinoma of the lung.)

*Group* 3.—Tumour from regional nodes implanted. No demonstrable organ metastases. (Carcinoma of breast. Carcinoma of naso-pharynx.)

*Group* 4.—Organ metastases implanted. (Squamous cell carcinoma of lung with liver metastases (surgical biopsy.) Lymphosarcoma in a child of 7. (Post-mortem material from liver.)

*Group* 5.—Centrifuged deposits from the following :

(1) Pleural fluid containing malignant cells in a case of squamous cell carcinoma of the cervix, proven at autopsy. (12 mice inoculated; 6 on cortisone.)

(2) Sternal marrow obtained after death from a case of acute leukaemia (monocytic type Naegli.)

(3) Sternal marrow from a case of Hodgkin's Disease.

Details of the three successful cases in which larger numbers of mice were used for implantation follow :

*Case F.*—Male, aged 50. Admitted to Harefield Hospital on September 17, 1952 with a history of lassitude since June, 1952. Signs of collapse of the left upper lobe with overlying pleurisy were present. Bronchoscopy revealed a mass of tissue blocking the left upper lobe orifice. Biopsy showed " oat cell " carcinoma. Radiotherapy was given as the growth was inoperable. Bronchoscopy on December 31, 1952, revealed no sign of the tumour and biopsy showed a few degenerate cells only. Patient returned in April, 1953, with six subcutaneous nodules, the largest of these was removed under local anaesthesia and revealed " oat cell " carcinoma.

Portions of this tumour were used for implantation. Patient died May 17, 1954, with generalised secondaries. No autopsy performed, but there were signs of secondary deposits in the brain.

*Case* C.—Male, aged 52. Admitted to Harefield Hospital under the care of Mr. T. Holmes Sellors with 7 weeks' history of pains in the chest and haemoptysis. X-ray revealed a mass in the left lower lobe apical section, and on September 3, 1953, a left pneumonectomy was performed. Recovery was uneventful, but on October 2, 1953, patient was re-admitted with secondary deposits and died on December 12, 1953.

The specimen consisted of a large peripheral type growth in the left lower lobe (apical section). Growth had spread to the broncho-pulmonary glands, but although the hilar glands were free, removal was probably incomplete, due to invasion of the pleura. Histology showed an " oat cell " carcinoma with tubule formation and extensive squamous metaplasia.

*Case J.*—Male, aged 56. Admitted to Edgware General Hospital June, 1953, under the care of Dr. G. S. C. Sowry with an opacity in the right lung. Biopsy showed an "oat cell" carcinoma. His condition improved on radio-therapy, but in October, 1953, he was admitted to the Middlesex Hospital with deposits in the spine and numerous subcutaneous nodules over the chest and abdomen. One of these was removed under local anaesthesia and used for implantation (Fig. 7). The patient died 4 days later. Permission for a post-mortem was refused.

The results of tumour transplantation in these three cases are conveniently summarized in Table II.

TABLE II.—*Results of Tumour Transplantation.*

| | Number of mice implanted. | Number surviving after 7 days. | Number of mice showing "microscopic tumours." | Number of mice showing "macroscopic tumours." |
|---|---|---|---|---|
| *Case F.* | | | | |
| Cortisone treated | 9 | 6 | 2 (13 & 26) | 0 |
| No cortisone | 12 | 10 | 0 | 1 (128) |
| *Case C.* | | | | |
| Cortisone treated | 6 | 5 | 1 (56) | 0 |
| No cortisone | 6 | 5 | 0 | 0 |
| *Case J.* | | | | |
| Cortisone treated | 12 | 12 | 1 (43) | 0 |
| No cortisone | 12 | 10 | 0 | 1 (66) |

The figures in brackets refer to the number of days at which growth was observed.

### HISTOLOGY.

*Site of growth.*

Growing tumour was found in the brain substance, in the ventricles, in the choroid plexus and in one cortisone-treated mouse in the stroma of the donor tissue when the implant was not wholly within the brain substance.

Murphy and Sturm (1923), discussing conditions determining the transplantability of tissues in the brain, and working with mouse tumours transplanted to the brain of rats, guinea-pigs and pigeons, found that the growth of foreign tissue in the brain took place only when the grafted material lay entirely in the brain substance, but when it came in contact with the ventricle a cellular reaction took place with resultant destruction of the graft.

Fig. 16 and 17: Case J shows vascularized invasive growth at 43 days in a cortisone-treated mouse replacing the ependymal lining of the lateral ventricle with no surrounding cellular response.

Fig. 18 and 19: Case F shows growth invading the brain substance from the ventricle of a cortisone-treated mouse dying at 13 days after inoculation of scrapings from the cut surface of the donor tumour.

No metastases were observed at post-mortem or on histological examination of liver, lungs, and kidneys of the mice where intracranial growth was observed. Intracranial transfer of portions of the macroscopic tumours to other mice was unsuccessful. There was a delay of some hours before transfer, as both mice died from the intracranial tumours.

*Type of growth, cellular reaction and vascular supply.*

In 1912 DaFano investigated homologous intracerebral transplantation of animal malignant tumours and found that growth was infiltrative or expansive, or a combination of both. The stroma originated from the blood supply of the brain and a reaction of plasma cells and lymphocytes appeared only in partially immune animals.

In the present investigation infiltrative and expansive growth was found, and the morphology of the cells resembled that of the original biopsy.

Although numerous thin-walled blood vessels could be seen in the tumours it is not obvious if these were provided by the host or grew from the implanted tissue. The macroscopic tumour of Case J (Fig. 5 and 6) was surrounded by an area containing blood. Numerous thin-walled vessels in the tumour opened into this pool of blood, but no direct continuity of cerebral and tumour vessels could be seen.

In the macroscopic tumour of Case F there was no surrounding pool of blood, but there were numerous vessels in the tumour and at the periphery adjacent to the brain substance (Fig. 9). The centre of this tumour was undergoing necrosis when the mouse died on the 128th day after implantation.

Vessels were seen in the growths at 56 days (Case C) and 43 days (Case J).

There was no cellular reaction around the macroscopic or the microscopic tumours at 56 days (Case C) and 43 days (Case J). At 26 days (Case F) there was a mild reaction round the graft of mononuclear cells, lymphocytes, microglial and gitter cells, and occasional plasma cells.

This cellular reaction was not as marked as that seen around some of the implanted tissue from cases of breast cancer and in two mice dying on the 11th day (Case F) where cortisone was not started until 5 days after implantation.

Portions of mouse hair and fragments of skull bone implanted with the tumour were sometimes observed (Fig. 10).

### DISCUSSION

One of the major difficulties of intracranial transplantation is in the choice of suitable material from the tissues submitted.

Franks (1953) transplanting animal tumours to the guinea-pig eye found that with solid tumour transplants there was almost always central necrosis and a very severe inflammatory reaction with a polymorphonuclear exudate. With cell suspensions or ascites tumour cells the initial inflammatory reaction was much less marked.

Greene (1953) reporting the successful heterologous transplantation of thirteen cases of lung cancer stated : " The point to be emphasized is that the incidence of 'takes' in the first-generation passage of a heterologous tumour reflects its content of desmoplasia and the experience of the operator rather than any essential character of the neoplastic cells. From this point of view a single 'take' is as expressive of the true nature of the tumour as a 100 per cent incidence and unless a complete uniformity of transplanted fragments with respect to desmoplasia and parenchyma is assured, a tabulation of the incidence of 'takes' is not subject to meaningful interpretation."

In the present series growth has been obtained by using each of the techniques outlined previously, but the original tumours in each case contained a large proportion of malignant cells relative to the supporting stroma.

The result in tumours containing more stroma can be illustrated in a case of carcinoma of the breast, where the fragments of tissue adjacent to those used for implantation showed roughly equal proportions of stromal and parenchymatous areas. One of the mice dying at 6 days showed the implanted material to consist of a mass of stromal fibrous tissue with no tumour cells in the portion sectioned (Fig. 20). Supporting tissue appeared to survive in the brain for a considerable number of days after implantation.

Some of the failures may be due to the fact that frozen sections were not done to assist in the choice of suitable material for implantation. In addition it was not always possible to locate the original implanted tissue or scrapings, and serial sections were not employed when nothing was seen in the operation area.

The survival rate of the mice was also a limiting factor. Only a few died during the operation, and these were replaced if enough tumour material was available. Some mice had to be killed or died in the first 14 days after implantation from trauma, haemorrhage, infection or hydrocephalus. After this somewhat stormy convalescence, no gross neurological signs were noticed except in one mouse (Case J), which showed paralysis of the left hind leg on the 59th day after intracerebral tumour inoculation. This mouse died 7 days later from the effect of the " human " tumour growing and compressing the brain and distending the skull.

Although it appears from the table that there is an increased incidence of microscopic growth in the cortisone-treated animals, the number of surviving animals was too small to assess the effects of the hormone on the growth of the implant or the cellular response of the host.

Material from cases of lung cancer appears to grow more readily than other tumours.

This has been the finding of Greene (1953), who postulates that human tumours which can be grown in animals are autonomous, whilst those which do not grow are in a dependent phase. The explanation may however be related to the host tissue rather than the tumour, and it is interesting that lung tumours have a peculiar tendency to metastasize to the brain.

In the three successful growths reported here, Case F died within 2 years of first admission to hospital, and Cases C and J within 6 months.

Case F and Case J had received radiotherapy before the appearance of the secondary subcutaneous nodules used for animal implantation.

To summarize, it is apparent that in view of the results obtained in these experiments and in those of other workers, heterologous transplantation using techniques at present available, does not appear to be a practical routine test for the biological activity of any given tumour, but it may provide a method for propagating certain human tumours in laboratory animals for experimental purposes.

### SUMMARY.

Material from 20 human tumours has been implanted intracranially into a series of 180 mice.

No growth of the implanted tissue could be demonstrated in 17 of the 20 groups of mice.

In the remaining 3 cases of metastatic " oat cell " carcinoma of the lung infiltrating growth was obtained between 13 and 128 days in 6 of the mice implanted.

One mouse each from 2 of these cases died 66 and 128 days after implantation from the effects of a large expanding intracranial vascularized "human" tumour. On section these occupied about half the width of the normal mouse skull and on microscopy showed malignant cells with numerous mitoses resembling the original biopsy.

Some of the animals were treated with cortisone in an attempt to increase the number of successful implants; but there is insufficient data on the small number of animals surviving to assess the effects of the hormone. Growth was obtained in animals treated with cortisone as well as in normal animals.

Part of the expenses of this investigation has been defrayed by the British Empire Cancer Campaign. Cortisone was received from the Medical Research Council and from Messrs. Merck and Company, Rahway, New Jersey, U.S.A.

I wish to express my thanks to the following: Professor R. W. Scarff and Dr. A. C. Thackray for their encouragement and help; Dr. J. B. Walter for many helpful suggestions and for providing some of the material used; to Dr. L. M. Franks for placing at my disposal his modification of the Bashford needle; to Mr. E. V. Willmott, F.R.P.S. for some of the photographs, and to Mr. H. A. Barker, Mr. E. C. Clayden and Mr. A. E. Cooper for unlimited and invaluable technical assistance.

### REFERENCES.

Andrus, S. B., Meissner, G. F. and Whorton, C. M.—(1951) *Cancer*, **4**, 1015.
Chesterman, F. C.—(1953) *Rep. Brit. Emp. Cancer Campgn*, **31**, 72.
DaFano, C.—(1912) *Folia neuro-biol.*, Band VI, Nr. 2 und 3, p. 109.
Ehrlich, P.—(1907) *Z. Krebsforsch.*, **5**, 59.
Eichwald, E. J., Goodman, G. J. and Chang, H. Y.—(1951) *Proc. Soc. exp. Biol., N.Y.*, **78**, 72.
Franks, L. M.—(1953) *J. Path. Bact.*, **65**, 275.
Geissler, T.—(1893) 'Arbeiten aus der chirug.' Klinik der Königlichen Universitat zu Berlin, 8te. Thiel., Berlin, p. 70.
Green, H. N. and Whiteley, H. J.—(1952) *Brit. med. J.*, ii, 538.
Greene, H. S. N.—(1938) *Science*, **88**, 357.—(1941) *J. exp. Med.*, **73**, 461 and 475.—(1942) *Cancer Res.*, **2**, 649.—(1946) *Yale J. Biol. Med.*, **18**, 239.—(1948) *J. Amer. med. Ass.*, **137**, 1364.—(1949) *Yale J. Biol. Med.*, **22**, 611.—(1951) *Cancer Res.*, **11**, 529.—(1952) *Cancer*, **5**, 24.—(1953) *Cancer Res.*, **13**, 347.—(1954) *Ibid.*, **14**, 516.
Kemler, R. L. and Graham, E. A.—(1950) *Cancer*, **3**, 735.
Lumb, G. D.—(1954*a*) *Brit. J. Cancer*, **8**, 442.—(1954*b*) *Rep. Imp. Cancer Res. Fd.*, **51**, 13.
Morris, D. S., McDonald, J. P. and Mann, F. C.—(1950) *Cancer Res.*, **10**, 36.
Murphy, J. B.—(1913) *J. exp. Med.*, **17**, 482.
*Idem* and Sturm, E.—(1923) *Ibid.*, **38**, 183.
Putnoky, J.—(1930) *Z. Krebsforsch*, **32**, 520.
Sailer, J.—(1900) *Amer. J. med. Sci.*, **120**, 190.
Selbie, F. R.—(1936) *Amer. J. Cancer*, **28**, 530.
Shirai, Y.—(1921) *Japan med. World*, **1**, No. 2, 14.
Toolan, H. W.—(1953) *Cancer Res.*, **13**, 389.
Towbin, A.—(1951) *Ibid.*, **11**, 716.
Woglom, W. H.—(1913) 'George Crocker Special Research Fund,' **1**, 50.